# Seeking a second medical opinion: composition, reasons and perceived outcomes in Israel

**DOI:** 10.1186/s13584-017-0191-y

**Published:** 2017-12-08

**Authors:** Liora Shmueli, Nadav Davidovitch, Joseph S. Pliskin, Ran D. Balicer, Igal Hekselman, Geva Greenfield

**Affiliations:** 1 Department of Health Systems Management, Ben-Gurion University of the Negev, P.O. Box 653, 84105 Beer-Sheva, Israel; 2 Department of Industrial Engineering and Management Ben-Gurion University of the Negev, P.O. Box 653, 84105 Beer-Sheva, Israel; 3 Clalit Research Institute, Clalit Health Services, 101 Arlozorov, 62098 Tel-Aviv, Israel; 4 Clalit Mushlam Health Insurance Systems, Clalit Health Services, 1 Ben Gurion, 5120149 Bnei Brak, Israel; 5 Department of Primary Care and Public Health, School of Public Health, Imperial College London, The Reynolds Building, St. Dunstan’s Road, London, W6 8RP UK

**Keywords:** Second opinion, Health seeking behaviors, Patient-physician communication, Health policy, Survey

## Abstract

**Background:**

Seeking a second-opinion (SO) is a common clinical practice that can optimize treatment and reduce unnecessary procedures and risks. We aim to characterize the composition of the population of SO seekers, their reasons for seeking a SO and choosing a specific physician, and their perceived outcomes following the SO.

**Methods:**

A cross-sectional national telephone survey, using a representative sample of the general Israeli population (*n* = 848, response rate = 62%). SO utilization was defined as seeking an additional clinical opinion from a specialist within the same specialty, for the same medical concern. We describe the characteristics of respondents who obtained SOs, their reasons for doing so and their perceived outcomes: (1) Satisfaction with the SO; (2) Experiencing health improvement after receiving a SO; (3) A difference in the diagnosis or treatment suggested in the first opinions and the second opinions; (4) Preference of the SO over the first one.

**Results:**

Most of the respondents who sought a SO (*n* = 344) were above 60 years old, secular, living with a partner, perceived their income to be above average and their health status to be not so good. For the patients who utilized SOs, orthopedic surgeons were sought out more than any other medical professional.Reasons for seeking a SO included doubts about diagnosis or treatment (38%), search for a sub-specialty expert (19%) and dissatisfaction with communication (19%). SO seekers most frequently chose a specific specialist based on a recommendation from a friend or a relative (33%). About half of the SO seekers also searched for information on the internet. Most of the respondents who sought a SO mentioned that they were satisfied with it (84%), felt health improvement (77%), mentioned that there was a difference between the diagnosis or treatment between the first opinion and the SO (56%) and preferred the SO over the first one (91%).

**Conclusions:**

Clinical uncertainty or dissatisfaction with patient-physician communication were the main reasons for seeking a SO. Policy makers should be aware that many patients choose a physician for a SO based on recommendations made outside the medical system. We recommend creating mechanisms that help patients in the complicated process of seeking a SO, suggest specialists who are suitable for the specific medical problem of the patient, and provide tools to reconcile discrepant opinions.

**Electronic supplementary material:**

The online version of this article (10.1186/s13584-017-0191-y) contains supplementary material, which is available to authorized users.

## Background

It is reasonable to expect multiple opinions for clinical cases. Inevitable discrepancies in clinical judgment make second opinions (SOs) clinically important [1–6] and cost-effective [7–9] due to their potential to reduce costs of unnecessary, expensive and invasive diagnostic and surgical procedures. People who face a crucial decision such as undergoing major surgery are likely to seek a SO.

### SO definition and regulation

There are three main types of SO: the first one reflects the patient’s desire to confirm the best diagnosis, treatment, or prognosis suggested by his first physician [10]. The second type, initiated by the physician, who is looking for the advice of a second specialist. The third type, related to SO programs usually imposed, on patients and doctors alike, by third party insurers as a cost containment measure (often referred to as prior authorization). SO programs were first introduced in the US in the 1970s by insurance companies as a pre-authorization tool before elective surgery. There are major differences among countries in health policy, access and payment mechanisms for SOs. Some states in the US (e.g., Florida, Indiana, Louisiana, Missouri, New Hampshire and New York) have passed laws in the 1990s to ensure the patient right for a SO [11]. Some plans in the US require a referral from the primary care physician, and require seeing an in-network physician [12]. In Canada, there is no mandatory SO requirement for surgery [13]. In some other countries in Europe it is not a formal right. For example, in the UK, patients do not have a legal right to a SO, although a healthcare professional will rarely refuse to refer them for one [14].

### Advantages and disadvantages of SOs

SOs have advantages and disadvantages in several aspects: clinically, the vast evidence on diagnostic discrepancies between independent first and SOs [1–6, 15] highlights the clinical importance of obtaining SOs. This is especially important because surgical judgment can differ radically from one surgeon to another [16–18], and many surgeries eventually appear to be unnecessary [19]. However, in cases where SOs stem from mere anxiety, common in difficult conditions, consulting many physicians for the same illness episode (a behavior called “Doctor shopping”) may lead to patient confusion and resources waste, especially when there is no informed reconciliation of conflicting opinions, and have a higher risk of in-hospital complications [20].

Financially, the aim of mandatory SO programs was based on the premise that they can reduce costs of unnecessary, expensive and invasive diagnostic and surgical procedures and save rehabilitation costs [8, 9, 21, 22]. Moreover, patients tend to adhere to a SO recommending non-invasive therapy rather than surgery, thus SOs can reduce the need for surgery by 50% and save costs [23]. Yet, in practice, many SOs are not part of organized programs and so, there is no organized mechanism. Therefore, SOs can be a financial burden both to patients and systems in the absence of a regulated mechanism. A US survey estimated the annual cost of SOs at $3.2 billion [11].

### Second opinions in the Israeli healthcare system

A detailed description of access to SOs in Israel appears elsewhere [24]. In Israel, patients are entitled to obtain SOs according to the Patient Rights Law (1995), but there is no explicit right to SO within Israel’s National Health Insurance system and no earmarked government allocation for SOs.

The Israeli healthcare system consists of four health funds providing primary and secondary care. The health funds also provide supplemental, voluntary health insurance schemes that provide partial reimbursement for out-of-pocket SO consultations, among other benefits. More than 75% of the population are covered by voluntary, supplementary health insurance provided by the health funds. In practice, people obtain SOs also through the secondary care provided by the health funds, through private insurance plans that provide reimbursement for out-of-pocket SO consultations, or by paying directly out of pocket to the private physician. Clearly, this situation discriminates against lower socio-economic patients who are not insured through either supplementary or commercial private insurances. Co-payments and limited access in the periphery create additional barriers to fulfill the right to SO as intended by the Patient Rights Law.

Currently, SOs in Israel are funded through a variety of mechanisms, with some funded through the universal NHI benefits package, others funded through voluntary (and non-universal) insurance programs operated by the health plans and the commercial insurance companies, and still others funded through out-of-pocket payments. Unfortunately, it is not clear what proportion of SOs is funded by each of these three sources, which differ substantially in their equity implications. What is clear is that currently there is no explicit right to SO within the basic benefits package.

The demand for SOs in Israel funded through health plan supplementary insurance programs increasing rapidly; the total net expenditure of the Israeli health funds’ supplementary insurances on SOs^1^ dramatically increased by 78.7% from 2006 to 2010 [25] The net expenditure on SOs in 2011 across supplementary health insurance provided by the health funds (after reducing income from co-payments) was equivalent to $93.4 million [25], which is the second largest expenditure after surgery, accounting for about 13% of the total expenditure of the supplemental health insurance provided by the health funds. Currently there is no policy regarding SOs as a tool for controlling surgical procedures or costs in Israel, and data are not available on SO influences on surgical expenses. The steep rise in utilization of SOs, part of the rise in acquisition of supplementary insurance programs [26], reflects the shift from pure private encounters to a private-public mix characterizing the supplementary insurance environment [27].

### Second opinion utilization in Israel compared to other countries

To the best of our knowledge, only a few studies have evaluated how many people actually seek SOs [28], and most of them surveyed patients with cancer. Only one study addressed this question in a general population, conducted 20 years ago, and showed that 18.8% of the US respondents obtained a SO [11]. A study conducted by us [29] using electronic claims data in Israel, showed that 15.0% of 1,395,816 people sought a SO, mostly from orthopedic surgeons [25]. Much higher rates were found in east-Asian countries [10, 30] and in Israel (45% in selected cancer patients) [31], but only 6.5% in Australia [32]. Even higher rates were found when patients were asked about their intention to seek a SO: 80% of 1513 US patients said that they are likely to seek a SO for a serious diagnosis as a safety precaution [33], and an independent breast cancer SO was desired by 94% of 617 German breast cancer patients [34] (See Additional file 1: Appendix 1).

The literature on patient-initiated SOs is limited [35], and only a few studies explored the reasons for seeking a SO and what influences patients while choosing a specific physician. The lack of up-to-date data on SO utilization motivated us to conduct a nationally representative survey regarding the use of SOs in the general Israeli population.

### Objectives

In a previous paper we reported findings on the frequency of SO utilization [29]. In this paper, we aim to characterize the population composition of SO seekers, their reasons for seeking a SO and choosing a specific physician and their perceived outcomes following the SO. Understanding patients’ reasons for seeking SOs and their perceptions of its impact, is important for two reasons. First, seeking SOs has consequences for expenditure, policy, clinical outcomes and satisfaction. Additionally, obtaining SOs reflects broader changes of consumerism and patient choice [36].

## Methods

### Design

The study is a part of a large mixed methods study (qualitative in-depth interviews, electronic medical records analysis and a telephone survey) aimed to explore the utilization of SOs, including access, inequalities, decision making, policy and patient-physician relationship. We conducted a cross-sectional national telephone survey asking people about their SO search behavior and their reasons for seeking a SO. The survey was conducted in collaboration with the B.I. and Lucile Cohen Institute for Public Opinion Research, an academic survey institute at Tel-Aviv University, during November 2011. The interviewers followed a predefined closed-end protocol (See Additional file 2: Appendix 2). Respondents were interviewed in their native language (Hebrew, Russian or Arabic). We followed the STROBE guidelines for reporting cross-sectional studies [37].

### Participants and sampling

We sampled a representative random sample of the general Israeli adult population. The inclusion criterion was being 18 years old and above. Respondents were sampled by a probabilistic sampling of households from layers of statistical areas, defined by socio-demographic characteristics of each area. Layers were designed to create homogeneity on the basis of geographic area (e.g., between large cities and small towns), immigration (native-born and established immigrants), level of religiosity (secular and orthodox) and socio-economic level. Sampling was done so that the probability of each statistical area to be included in the sample is proportional to the size of the population in the area. Such sampling ensures representation of various population groups, particularly those with a relatively small proportion. The minimal required response rate was predetermined to be 50%. The sample size was based on a pre-test conducted with 274 respondents, which showed that about 20% of them had obtained a SO.

**Fig. 1:**
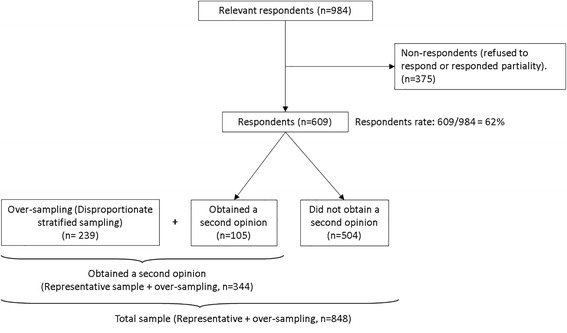
Sample selection

We used disproportionate stratified sampling to increase the number of respondents who obtained a SO for the inferential statistics. This method allows different sampling ratios in different strata. This permits heavier sampling in subgroups with few members, to provide acceptable estimates not only for the population as a whole, but for each of its subgroups [38]. The purpose of the disproportionate stratified sampling was to ensure there are at least 300 respondents who obtained a SO. We over-sampled another 239 respondents who obtained a SO, using the same principles of sampling layers of statistical areas as the representative sample. Hence, the survey included a total of 848 people from the representative sample and the disproportionate stratified sample (Fig. 1). The study was approved by the Institutional Ethics Committee for non-clinical studies (Approval K2010/137).

### Variables and measurements

The dependent binary variable was self-reported SO utilization. A ‘SO’ was defined as ‘*consulting with another specialist, in the same specialty, in order to obtain a SO on the same medical concern during the past 12 months (excluding consultations with family physicians’,* as our definition is related to secondary care and not primary care.

The covariates were: (1) age; (2) gender; (3) education level; (4) personal status (in partnership or not); (5) ethnicity; (6) level of religiosity; (7) self-reported income level; (8) socioeconomic level, based on the Israeli Central Bureau of Statistics; (9) being an immigrant (defined as immigration to Israel after 1989); (10) country of birth; and (11) perceived health status.

### Statistical analyses

We chose the respondents who sought a SO (representative sample = 105 and over-sampling = 239, for a total of 344) for the inferential analyses.

We describe the characteristics of respondents who obtained a SO and their following perceived outcomes:

1. Satisfaction with the SO: “To what extent were you satisfied with the second opinion consultation?” (Question #13 in the protocol, see Additional file 2: Appendix 2).
2. Experiencing health improvement after getting the SO: “To what extent did you feel an improvement in your health condition following the second opinion consultation?” (Question #14 in the protocol, see Additional file 2: Appendix 2).
3. A difference in the diagnosis or treatment suggested in the firstopinions and the second opinions: “Was there a difference in diagnosis or treatment between the two specialists?” (Question #11 in the protocol, see Additional file 2: Appendix 2).
4. Preference of the SO over the first one: “Which opinion did you choose?” (Question #12 in the protocol, see Additional file 2: Appendix 2).

We described the participants’ entitlement to seeking a SO with the question: “Are you aware of your right to seek a second medical opinion on a medical concern?” (Question #1 in the protocol, see Additional file 2: Appendix 2). We present only the descriptive statistics, because after conducting the univariate analysis we realized that the samples in the outcome questions by socio-demographic characteristics were too small for univariate analyses.

We explored the participants’ reasons for seeking a SO with the question: “What were your reasons for seeking a second medical opinion?” (Question #9 in the protocol, see Additional file 2: Appendix 2).

We explored the participants’ process of selecting the specific specialist with the question: “What made ​​you choose the specialist from which you obtained the second opinion?” (Question #10 in the protocol, see Additional file 2: Appendix 2).

We explored whether the participants consulted with a source outside the medical system with the question: “Did you consult one of the following in parallel to seeking a second medical opinion, regarding the same problem?” (Question #15 in the protocol, see Additional file 2: Appendix 2).

## Results

We approached 984 households, out of which 609 questionnaires were completed in full (response rate 62%). 105 respondents from the representative sample (out of 609) visited a physician for a SO during the study period. With the disproportionate stratified sampling, a total of 344 respondents obtained a SO (an addition of 239 to the 105 respondents from the representative sample). While almost all of those who sought a SO knew about their entitlement to seek a SO (92%), only 75% of those who did not seek a SO knew about this entitlement (χ2 = 40.5 (2), *p* < .001). Hence there are 25% of the non-seekers who could potentially benefit from a SO, if they knew they were entitled to get one.

Descriptive characteristics of the respondents who sought a SO are shown in Table 1. Most of them were female, above 60 years old, had an academic education, were living with a partner, were secular Jews, and were native-born Israelis or established immigrants. They perceived their health status to be not so good, perceived their income to be above average and were classified as middle and high socioeconomic level.

**Table 1:** Characteristics of respondents who sought a SO (*n* = 344)

Characteristics	Sought a second opinion (*n* = 344)
Gender	
Male	137 (35.8%)
Female	207 (44.5%)
Age group	
18–39	98 (38.1%)
40–59	120 (37.7%)
60+	126 (46.2%)
Education	
Basic	43 (35.5%)
High school	157 (37.5%)
Academic	143 (46.7%)
Missing values	1
Personal status	
Living with a partner	273 (42.7%)
Not living with a partner	69 (34.2%)
Missing values	2
Ethnicity	
Jewish	285 (41.1%)
Non-Jewish	54 (36.7%)
Missing values	5
Religiosity	
Religious	115 (39.5%)
Secular	224 (41.0%)
Missing values	5
Self-reported income group	
Well below the average	84 (39.1%)
Around the average	183 (37.6%)
Well above the average	28 (50.0%)
Missing values	49
Socio-economic level (by residential area)	
Low	68 (33.7%)
Middle	262 (42.7%)
High	6 (46.2%)
Missing values	8
Immigration	
Native-born and established immigrants	312 (41.5%)
New immigrants	32 (33.3%)
Country of birth	
Israel	220 (39.9%)
Europe/America	37 (47.4%)
Soviet Union	29 (29.6%)
Asia/North-Africa	56 (47.5%)
Missing values	2
Perceived Health status	
Very good	128 (31.9%)
Good	131 (42.4%)
Not so good	80 (62.5%)
Missing values	5

Note: Percentages are calculated as valid % per each row (i.e., each row sums up to 100%, without missing values)

*p < 0.05

### Specialties approached for a SO

Almost a third of the respondents sought a SO from an orthopedic surgeon. Other common specialties were ophthalmology, gynecology, general surgery, and ear, nose and throat (ENT) (Table 2).

**Table 2:** Distribution of second medical opinion visits by specialty

Specialty	N (%)
Orthopedics	111 (32.3%)
Ophthalmology	31 (9.0%)
Gynecology	28 (8.1%)
Ear, Nose and Throat	21 (6.1%)
General Surgery	21 (6.1%)
Dermatology and venereology	20 (5.8%)
Pediatrics	18 (5.2%)
Cardiology	17 (4.9%)
Oncology	10 (2.9%)
Neurology	10 (2.9%)
Other specialties	57 (16.6%)
Total	344 (100.0%)

### Reasons for seeking a SO

The most common reasons for seeking a SO were to verify a diagnosis made by the first specialist physician or doubts about the treatment recommended (38%). Other reasons were seeking an opinion from a sub-specialist for the specific condition (19%) or dissatisfaction with communication with the first physician or feeling that the physician did not provide enough information (19%). Respondents also sought a SO because previous treatments were ineffective, or for other reasons. Because the respondents could provide more than one reason for seeking a SO, the number of reasons is larger than the number of respondents (Table 3).

**Table 3:** Reasons for seeking a second opinion (*n* = 422 reasons provided by the 344 respondents who obtained a second opinion)

Reasons for seeking a second opinion	n (%)
I wanted to test the suggested diagnosis with another doctor; I had \doubts about the treatment recommended	161 (38.1%)
I was looking for sub-specialist	82 (19.4%)
I was unsatisfied from the communication with the doctor, there was no “chemistry”; I felt they didn’t give me enough information and I want a detailed explanation.	81 (19.2%)
The previous treatment was ineffective	65 (15.4%)
Other reasons	33 (7.8%)

Respondents could provide more than one reason for seeking a second opinion. Hence, the number of reasons is larger than the number of respondents

### Process of selecting a specific second opinion specialist

Almost a third of the SO seekers chose the specialist according to a recommendation they received from a friend or relative (115 of 344) and some chose the physician based on information on the internet (8%). Other methods of selecting the SO specialist were a recommendation from the family doctor (17%), a recommendation from another consultant physician (11%) and a recommendation from the service call center (10%) (Table 4).

**Table 4:** Reasons for choosing a specific second opinion physician

Reasons for choosing a specific second opinion physician	*n* = 344 (%)	
Recommendation from a friend or a relative	115	(33.4%)
Recommendation from the family doctor	58	(16.9%)
Recommendation from another consultant physician	36	(10.5%)
Recommendation from the service call center or secretary	34	(9.9%)
Information on the internet	27	(7.8%)
Previous acquaintance with the doctor within the private system	25	(7.3%)
Previous acquaintance with the doctor within the public system	10	(2.9%)
Independent decision- by proximity and availability	12	(3.5%)
Reputation of the physician	8	(2.3%)
Other	19	(5.5%)
Total	344	(100.0%)

### Consulting outside the health system in parallel to seeking a second medical opinion for the same problem

About half of the respondents who sought a SO (160 of 344) mentioned that they also searched an alternative advice outside the health system in parallel to seeking a second medical opinion, for the same problem. Most of them searched for information on the internet (47%), alternative medicine (30%) or a Rabbi (12%).

### Perceived outcomes following the second opinion

Most of SO seekers mentioned they were satisfied with the SO (84% out of 332), experienced health improvement after receiving the SO (77% of 298), mentioned there was a difference between the diagnosis or treatment between the first opinions and the SOs (56% of 305) and preferred the SO over the first one (91% of 177), (Table 5).

**Table 5:** Perceived outcomes following the second opinion

Perceived outcomes of getting a second opinion		*n* = 344 (%)	
I was satisfied with the SO	Not satisfied	52	(15.7%)
	Satisfied	280	(84.3%)
	Total	332	(100.0%)
	Missing values	12	
I experienced improvement after receiving the SO	Improved	228	(76.5%)
	Not improved	70	(23.5%)
	Total	298	(100.0%)
	Missing values	46	
There was a difference between the diagnosis or treatment between the first and the SO	Yes	171	(56.1%)
	No	134	(43.9%)
	Total	305	(100.0%)
	Missing values	39	
I preferred the SO over the first one	First	16	(9.0%)
	Second	161	(91.0%)
	Total	177	(100.0%)
	Missing values	167	

## Discussion

We performed the first survey of patient perceptions on SO utilization in Israel. This paper deals with a very important topic within the general field of medical decision making while seeking to balance quality of care and patient experience. The main findings from the present survey: A description of the population composition of SO seekers, their reasons for seeking a SO and for choosing a specific physician, and their perceived outcomes following the SO.

### Why do people seek SOs?

While many respondents obtained SOs for ‘clinical’ reasons (doubts about the recommended diagnosis or treatment, or search for an expert in a sub-specialty), many of them sought a SO because of dissatisfaction with patient-physician communication. This finding is well-documented in the literature: People also seek SOs when they are dissatisfied with an impersonal communication or feel they did not get enough information [4, 11, 30, 32, 34, 39–44]. The literature also shows that people mostly seek SOs when they believe a physician recommended a treatment other than what they believed was necessary, seek additional information or reassurance [32, 43, 45], or wish to ascertain whether the treatment is appropriate for them [10, 34, 42]. SOs are common after hearing a diagnosis of a serious illness [4], In many cases, people seek a SO simply because they hope that the diagnosis would turn out to be wrong [4, 44], are anxious [4, 30], or find it difficult to emotionally deal with an unexpected diagnosis [43, 45]. Hence, they would probably consult with a senior, well-known sub-specialist [46].

Many SOs were sought particularly from orthopedic surgeons, a finding consistent with previous literature [17, 18, 44]. Surgery may lead to subsequent complications and complex rehabilitation, yet delaying a necessary surgery may have deleterious effects, requiring even more radical intervention. SOs are hence common in orthopedics [18, 44] and general surgery [17]. Patients tended to adhere to a SO recommending non-invasive therapy rather than surgery, thus SOs can reduce surgery by 50% [23]. Another notable finding is that 3% of the respondents sought a SO from an oncologist, a fairly high rate, relative to the proportion of cancer in the Israeli population (1.6%) [47]. Indeed, seeking a SO is rather common among cancer patients [10, 31, 32, 48, 49].

### Process of selecting a specific second opinion specialist

Our research findings show that in many cases patients do not necessarily receive recommendations for choosing a specific medical specialist from whom to seek a SO, but rather base their choice on word-of-mouth from friends or relatives, or by searching for information on the internet. This finding is supported by previous studies showing that word-of-mouth and physician referrals were the primary sources of information for patients [50, 51]. Likewise, a recent systematic review showed that most patients rely on word-of-mouth recommendations while choosing a surgeon [52]. Additionally, some of the patients chose the physician based on information they received on the Internet, which is another form of getting an electronic word-of-mouth recommendation by other people [53]. Attention should be paid to this form of word-of-mouth information, especially with the increase of social media platforms and physicians should be aware of online reviews and their use by patients [54]. Professional and objective information about physicians on health care services websites can guide patients for choosing the right physicians by their specialty and work experience.

### Reasons for choosing an alternative source outside the medical system

Our findings show the phenomenon of consulting outside the health system in parallel to seeking a SO for the same problem. The finding that about half of the patients who sought a SO also consulted with an alternative source outside the health system (half of them on the Internet) is in line with the literature, emphasizing that many patients use the Internet for medical information [55]. They search information regarding their medical problem and possible treatments, and they consult with other patients or doctors. Searching data on the Internet cannot be a substitute for consultation with a physician who possesses all the historical and clinical information and clinical judgement. The finding that about 12% of the patients who sought a SO consulted with a Rabbi should be examined more deeply from the patients’ perspective. On the one hand it seems to be a small number, yet on the other hand, from the literature, the phenomenon of consulting with a Rabbi in parallel with the clinical consultation seems to be common in Israel, especially by physician assessments [24, 56].

### Policy implications and recommendations

The demand for SOs in Israel is constantly increasing with rising costs both to patients and the systems providing them. A crucial policy question is how this growing demand will be met under the current financial constraints faced by many health organizations [36]. These constraints derive from regulatory guidelines regardless of the payment method. In fee-for-service systems, SOs generate revenue to specialists regardless of whether they have changed the clinical decision. However, regulatory guidelines may limit the amount of consultations. In prepaid or capitated systems, such as in the European national health insurance systems, SOs do not generate revenue, hence insurers may apply gate-keeping policies on when and how they are used. Such gate-keeping, however, may collide with consumers’ desire for more information and choice. Even if a SO was not a legal right, people can still get a private SO if they can afford it, which may exacerbate health disparities in a manner similar to any other private medical service. In some states in US, the right for SO was hence stated by law [11].

In Israel, the right for SO is stated by law, but the law does not say anything about who should pay for SOs. As SO is part of the patients’ health rights, there is a need to ensure its funding within the Israeli National Health Insurance Law, similar to other basic health services included in the national public healthcare basket. Current arrangements, where SO is covered only by supplementary insurance, create a situation where those not insured, or those insured that cannot afford co-payments, cannot enjoy SO options. Moreover, the supplementary insurance policies in Israel are at the border between the private and the public systems. The government does not allow premiums to be raised so that policyholders will be able to receive a second opinion in which the physician is paid at a rate that is competitive with the full out-of-pocket payment to a private physician.

In many cases SOs stem from dissatisfaction from communication with the first physician or feeling that the physician did not provide enough information. Thus better communication might reduce unnecessary SOs through improved patient satisfaction, answering patients concerns during the first consultation, hence saving costs for both the patient and the insurer. For example, if the surgeon explained thoroughly his/her reasons for advising a more invasive surgery, SO seeking could have also been reduced, and thus health resources could have been used for better purposes. The main question hence, is not whether too many or too little patients seek SOs – the question is whether those who can benefit from it can access it, and to ensure people get the right information they need in the first consultation through improved patient-physician communication.

SOs may also strain the trust relationship between the patient and the physician [41]. Without an informed reconciliation mechanism, patients may end up even more confused and unable to make an informed choice between the two opinions. Hence SOs may end up with increased health spending where clinical management is left unaffected or affected improperly. We recommend creating two kinds of mechanisms. The first one, a regulatory mechanism that helps patients in the complicated process of seeking a SO, refers people seeking a SO to specialists who are suitable for the specific medical problem of the patient and provides informed choice, thus reducing frustration. In the second feasible mechanism, to reconcile discrepant opinions, the SO can be a partial solution to fragmented care, when patients seek SO as a “stop-shop” after meeting different professionals and striving to reach a final decision. One can define a multidisciplinary consulting system. We have previously shown that these two mechanisms are lacking [41]. In sum, it is important to balance the pros and cons of SOs, weighing the patient’s benefit and efficient use of health resources.

### Limitations

The main limitation of the study stems from the definition of a SO as consulting another specialist within the same specialty. Patients may seek SOs from specialists in different specialties (e.g., an orthopedic surgeon and a neurologist for a backpain concern). They may also consult with a specialist for a SO on their primary care opinion. Hence our definition is conservative and might underestimate the volume of SOs. We chose this definition after thorough methodological considerations, to avoid misinterpretation of the question. Second, as in any survey, selection and recall biases may have occurred, as well as embarrassment and social desirability, as respondents might feel uncomfortable to disclose health conditions in a telephone survey.

## Conclusions

This study provides up-to-date survey data on SO utilization from the patient’s perspective. Providing data on SO utilization and exploring patients’ reasons for doing so and their reasons for choosing the specific SO physician are important for health policy makers and healthcare providers due to the consequences for expenditure, policy, clinical outcomes, and satisfaction. According to this study, the patients mentioned they had sought SOs due to doubts about the recommended diagnosis or treatment, but also due to dissatisfaction with patient-physician communication. Hence, many SOs can be potentially avoided by improved communication. Other aspects of choosing the SO and getting medical information, which affect medical decision making, should be taken into account as many patients choose a SO physician following recommendations outside the medical system, and also searched for information not necessarily using proper clinical means.

It is important to help patients in the complicated process of choosing SO and refer people seeking a SO to specialists who are suitable for the specific medical problem of the patient and to provide mechanisms to reconcile discrepant opinions. It is also essential to know whether SOs help them to get the right information they need, to help them make the right decision for them, and relieve them from the anxiety they experience. Still, patients and providers do not have appropriate tools for deciding regarding the SO. Appropriate tools should address the complexity of making rules about access and payment for SOs. Some questions still remain open: why do some people search on the Internet and do not seek a SO from a second physician? how to set a mechanism for SO, which will take into account aspects of costs, access, clinical and behavioural complements, in a way that will not produce inequalities and will not impair quality of care?

Further research is suggested to examine the cost-benefit of obtaining SOs and to gain knowledge about what patients and society get from SOs. It would also be useful to examine the frequency of use, composition of users, and perceived outcomes when a broader definition of “second opinions” is employed, to include visits to specialists in different specialties, for the same problem. These additional studies could contribute to informed policy decisions, balancing the patient benefit and efficient use of health resources.

## Additional files

Additional file 1: Appendix 1. Studies on second-opinion utilization rates. (DOC 72 kb)

Additional file 2: Appendix 2. A second medical opinion survey. (DOC 54 kb)

## Abbreviation

SOs: Second Opinions

## Footnotes

1 The financial reports of the health plans’ supplemental insurance programs include a line item entitled “second opinions”, but it is important to keep in mind that this term is somewhat misleading as the amounts presented include all payments to physicians for ambulatory care services, including first opinions.

## References

[1] Althabe F, Belizan JM, Villar J, Alexander S, Bergel E, Ramos S (2004). Mandatory second opinion to reduce rates of unnecessary caesarean sections in Latin America: a cluster randomised controlled trial. Lancet.

[2] Briggs GM, Flynn PA, Worthington M, Rennie I, McKinstry CS (2008). The role of specialist neuroradiology second opinion reporting: is there added value?. Clin Radiol.

[3] Kronz JD, Westra WH (2005). The role of second opinion pathology in the management of lesions of the head and neck. Curr Opin Otolaryngol Head Neck Surg.

[4] Mellink WA, Dulmen AM, Wiggers T, Spreeuwenberg PM, Eggermont AM, Bensing JM (2003). Cancer patients seeking a second surgical opinion: results of a study on motives, needs, and expectations. J Clin Oncol Off J Am Soc Clin Oncol.

[5] Wieske L, Wijers D, Richard E, Vergouwen MDI, Stam J (2008). Second opinions and tertiary referrals in neurology: a prospective observational study. J Neurol.

[6] Zan E, Yousem DM, Carone M, Lewin JS (2010). Second-opinion consultations in neuroradiology. Radiology.

[7] Rosenberg SN, Allen DR, Handte JS, Jackson TC, Leto L, Rodstein BM (1995). Effect of utilization review in a fee-for-service health insurance plan. N Engl J Med.

[8] Ruchlin HS, Finkel ML, McCarthy EG (1982). The efficacy of second-opinion consultation programs: a cost-benefit perspective. Med Care.

[9] McCarthy EG, Finkel ML, Ruchlin HS (1981). Second opinions on elective surgery. The Cornell/New York hospital study. Lancet.

[10] Tam KF, Cheng DK, Ng TY, Ngan HY (2005). The behaviors of seeking a second opinion from other health-care professionals and the utilization of complementary and alternative medicine in gynecologic cancer patients. Support. Care cancer off. J. Multinatl. Assoc. Support Care Cancer.

[11] Wagner T, Wagner L (1999). Who gets second opinions?. Health Aff Proj Hope.

[12] Medicare.gov M go. Second opinions before surgery [Internet]. 2017 [cited 2017 Jun 13]. Available from: https://www.medicare.gov/what-medicare-covers/part-b/second-opinions-before-surgery.html.

[13] Ridic G, Gleason S, Ridic O (2012). Comparisons of health Care Systems in the United States, Germany and Canada. Mater. Socio-Medica.

[14] NHS Choices. How do I get a second opinion? - Health questions - NHS Choices [Internet]. 2012 [cited 2013 Feb 18]. Available from: http://www.nhs.uk/chq/Pages/910.aspx?CategoryID=68&SubCategoryID=156.

[15] Lysack JT, Hoy M, Hudon ME, Nakoneshny SC, Chandarana SP, Matthews TW (2013). Impact of neuroradiologist second opinion on staging and management of head and neck cancer. J. Otolaryngol. Head Neck Surg.

[16] Rutkow IM (1982). Surgical decision making. The reproducibility of clinical judgement. Arch. Surg. Chic. Ill 1960.

[17] Grafe WR, McSherry CK, Finkel ML, McCarthy EG (1978). The elective surgery second opinion program. Ann Surg.

[18] McCarthy EG, Finkel ML (1981). Second consultant opinion for elective orthopedic surgery. Am J Public Health.

[19] Leape LL (1989). Unnecessary surgery. Health Serv Res.

[20] Chang H-R, Yang M-C, Chung K-P (2013). Can cancer patients seeking a second opinion get better care?. Am J Manag Care.

[21] Martin SG, Shwartz M, Whalen BJ, D’Arpa D, Ljung GM, Thorne JH (1982). Impact of a mandatory second-opinion program on medicaid surgery rates. Med Care.

[22] Gertman PM, Stackpole DA, Levenson DK, Manuel BM, Brennan RJ, Janko GM (1980). Second opinions for elective surgery. The mandatory Medicaid program in Massachusetts. N Engl J Med.

[23] Graboys TB, Headley A, Lown B, Lampert S, Blatt CM (1987). Results of a second-opinion program for coronary artery bypass graft surgery. JAMA. J Am Med Assoc.

[24] Greenfield G, Pliskin JS, Wientroub S, Davidovitch N (2012). Orthopedic surgeons’ and neurologists’ attitudes towards second opinions in the Israeli healthcare system: a qualitative study. Isr. J. Health Policy Res..

[25] Israeli Ministry of Health IM of. A public report on supplementary health programs of the Israeli Health Funds in 2011 [Internet]. 2011 [cited 2013 Feb 23]. Available from: https://www.health.gov.il/PublicationsFiles/sbn2011_17122012.pdf.

[26] Israeli Ministry of Health IM of H. A public report on supplementary health programs of the Israeli Health Funds in 2014 [Internet]. 2015. Available from: https://www.health.gov.il/PublicationsFiles/shaban2014_03012016.pdf.

[27] Filc D, Davidovitch N (2016). Rethinking the private–public mix in health care: analysis of health reforms in Israel during the last three decades. J. Health Serv. Res. Policy..

[28] Vashitz G, Davidovitch N, Pliskin JS (2011). Second medical opinions. Harefuah.

[29] Shmueli L, Shmueli E, Pliskin JS, Balicer RD, Davidovitch N, Hekselman I, et al. Second medical opinion: utilization rates and characteristics of seekers in a general population. Med Care. 2016;1 10.1097/MLR.0000000000000567 27213545

[30] Sato T, Takeichi M, Hara T, Koizumi S (1999). Second opinion behaviour among Japanese primary care patients. Br. J. Gen. PractJ R Coll Gen Pract.

[31] Sapir R, Catane R, Kaufman B, Isacson R, Segal A, Wein S (2000). Cancer patient expectations of and communication with oncologists and oncology nurses: the experience of an integrated oncology and palliative care service. Support Care Cancer.

[32] Tattersall MHN, Dear RF, Jansen J, Shepherd HL, Devine RJ, Horvath LG (2009). Second opinions in oncology: the experiences of patients attending the Sydney cancer Centre. Med J Aust.

[33] National Patient Safety Foundation. Public opinion of patient safety issues [Internet]. 1997 [cited 2012 Apr 30]. Available from: http://c.ymcdn.com/sites/www.npsf.org/resource/collection/abab3ca8-4e0a-41c5-a480-6de8b793536c/Public_Opinion_of_Patient_Safety_Issues.pdf?hhSearchTerms=%22Public+and+opinion+and+patient+and+safety+and+issues%22.

[34] Oskay-Ozcelik G, Lehmacher W, Konsgen D, Christ H, Kaufmann M, Lichtenegger W (2007). Breast cancer patients’ expectations in respect of the physician-patient relationship and treatment management results of a survey of 617 patients. Ann Oncol.

[35] Payne VL, Singh H, Meyer AND, Levy L, Harrison D, Graber ML (2014). Patient-initiated second opinions: systematic review of characteristics and impact on diagnosis, treatment, and satisfaction. Mayo Clin Proc.

[36] Saltman RB (2012). Viewing second opinions in terms of recent developments in patient choice. Isr J Health Policy Res.

[37] von Elm E, Altman DG, Egger M, Pocock SJ, Gøtzsche PC, Vandenbroucke JP (2007). The strengthening the reporting of observational studies in epidemiology (STROBE) statement: guidelines for reporting observational studies. PLoS Med.

[38] Abramson JH (2008). Research methods in community medicine: surveys, epidemiological research, programme evaluation, clinical trials. 6th ed. Chichester, England.

[39] Clauson J, Hsieh YC, Acharya S, Rademaker AW, Morrow M (2002). Results of the Lynn sage second-opinion program for local therapy in patients with breast carcinoma. Changes in management and determinants of where care is delivered. Cancer.

[40] Goldman RE, Sullivan A, Back AL, Alexander SC, Matsuyama RK, Lee SJ (2009). Patients’ reflections on communication in the second-opinion hematology-oncology consultation. Patient Educ Couns.

[41] Greenfield G, Pliskin JS, Feder-Bubis P, Wientroub S, Davidovitch N (2012). Patient-physician relationships in second opinion encounters - the physicians’ perspective. Soc Sci Med.

[42] Moumjid N, Gafni A, Bremond A, Carrere MO (2007). Seeking a second opinion: do patients need a second opinion when practice guidelines exist?. Health Policy Amst Neth.

[43] Philip J, Gold M, Schwarz M, Komesaroff P (2010). Second medical opinions: the views of oncology patients and their physicians. Support Care Cancer.

[44] van Dalen I, Groothoff J, Stewart R, Spreeuwenberg P, Groenewegen P, van Horn J (2001). Motives for seeking a second opinion in orthopaedic surgery. J Health Serv Res Policy.

[45] Sutherland LR, Verhoef MJ (1989). Patients who seek a second opinion: are they different from the typical referral?. J Clin Gastroenterol.

[46] Bayliss R (1988). Second opinions. Br. Med. J. Clin Res.

[47] Israeli Central Bureau of Statistics. Table 15.1. Persons reporting cancer, by sex and socio-demographic characteristics. [Internet]. 2009 [cited 2013 Feb 28]. Available from: http://www.cbs.gov.il/publications13/health_survey09_1500/pdf/t15_1.pdf.

[48] Hewitt M, Breen N, Devesa S (1999). Cancer prevalence and survivorship issues: analyses of the 1992 National Health Interview Survey. J Natl Cancer Inst.

[49] Morrow M, Jagsi R, Alderman AK, Griggs JJ, Hawley ST, Hamilton AS (2009). Surgeon recommendations and receipt of mastectomy for treatment of breast cancer. JAMA. J Am Med Assoc.

[50] Hanna N, Shoenbachler DD, Gordon GL (1995). Physician choice criteria. Health Mark Q.

[51] Dobele A, Lindgreen A (2011). Exploring the nature of value in the word-of-mouth referral equation for health care. J Mark Manag.

[52] Yahanda AT, Lafaro KJ, Spolverato G, Pawlik TMA (2016). Systematic review of the factors that patients use to choose their surgeon. World J Surg.

[53] Hennig-Thurau T, Gwinner KP, Walsh G, Gremler DD (2004). Electronic word-of-mouth via consumer-opinion platforms: what motivates consumers to articulate themselves on the internet?. J Interact Mark.

[54] Xu Y, Armony M, Ghose A. The Interplay between Online Reviews and Physician Demand: An Empirical Investigation. Rochester, NY: Social Science Research Network; 2016 May. Report No.: ID 2778664. Available from: https://papers.ssrn.com/abstract=2778664.

[55] Diaz JA, Griffith RA, Ng JJ, Reinert SE, Friedmann PD, Moulton AW (2002). Patients’ use of the internet for medical information. J Gen Intern Med.

[56] Keshet Y, Liberman I (2014). Coping with illness and threat: why non-religious Jews choose to consult rabbis on healthcare issues. J Relig Health.

