# Supplementary material for: Seeking a second medical opinion: composition, reasons and perceived outcomes in Israel
Source: Isr J Health Policy Res. 2017 Dec 8;6:67. doi: 10.1186/s13584-017-0191-y (PMC5721599; doi:10.1186/s13584-017-0191-y)
Supplement: Supplementary file 1 — Studies on second-opinion utilization rates. (DOC 72 kb) [file 13584_2017_191_MOESM1_ESM.doc]

**Appendix 1:** Studies on second-opinion utilization rates

| Authors | Domain | Second-opinion utilization rate | Characteristics of patients with greater likelihood of seeking a second opinion. | Country |
| --- | --- | --- | --- | --- |
| (Shmueli et al., 2016) | Orthopedics, ophthalmology, dermatology, ear, nose and throat (ENT), cardiology, general surgery, urology, gastroenterology, and neurology | 15.0% of 1,395,816 people (denominator-received first opinion from a relevant specialist) | - Women, native-born and established immigrants, people living in central urban areas or close to central urban areas, people with chronic conditions, and those who perceived their health status as not very good | Israel |
| (Sutherland & Verhoef, 1989) | Gastroenterology | 7.5% of 246 patients (denominator-received first opinion from a relevant specialist) | - Having symptoms for >2 years - Perceive their health to be fair to poor - Have seen <2 general practitioners in the past year - Have spent >6 days in a hospital during the last year | Canada |
| (Sutherland & Verhoef, 1994) | Gastroenterology | 16% of 341 patients (denominator-received first opinion from a relevant specialist) | - Seeking a second opinion was negatively related to internal locus of control, perceived health status, and demanding to know all details of treatment | Canada |
| (Hewitt, Breen, & Devesa, 1999) | Cancer | 55.7% of 659 patients had obtained a second opinion or multiple opinions (denominator- the general population) | - Hispanic origin - Patients with breast cancer (relative to other cancers) - Residents of the Northeast of the US; Patients living in non-central cities or suburbs of central cities (relative to those living in rural areas) - Gender, age at diagnosis, years since diagnosis, educational attainment, and level income had no statistically significant effect | US |
| (Wagner & Wagner, 1999) | General population | 18.8% of 3,789 patients (denominator-received first opinion from a relevant specialist) | - **Gender**: Female more than male - **Ethnicity/race**: White non-Hispanic patients sought more second opinions relative to Hispanics. Respondents who felt they would have received better care if they were of a different race were more likely to get an second opinion - **Satisfaction from care**: Persons who reported being treated badly (relative to those satisfied with treatment) - **Education**: Persons with a college degree (relative to less than a high school education) - **Insurance coverage**: HMO enrollees with private insurance (relative to HMO enrollees with public insurance). Patients not in an HMO obtained more SO than those enrolled in an HMO - Having a health problem - Hospital admission in the last year - Age, marital status, overall self-reported health status, household income, having a regular doctor, English as the primary language, and generation in United States had no significant effect. | US |
| (Sato, Takeichi, Hara, & Koizumi, 1999) | A sample of patients who visited a general medicine clinic at a medical school hospital | 40.6% of 1,033 patients had previously visited one medical facility (denominator- the general population) | - Living far from a medical school hospital - Anxious about their illness - Came to a university-affiliated hospital as a result of advice from anybody - No differences between the two groups related to gender, age, occupation, education, and marital status | Japan |
| (Sapir et al., 2000) | Cancer | 45% of 103 patients | - Not reported | Israel |
| (Tam, Cheng, Ng, & Ngan, 2005) | Cancer | 41.9% of 191 patients (denominator-received first opinion from a relevant specialist) | - Late-stage disease - Treatment with radiotherapy - Tertiary education | Hong-Kong |
| (Morrow et al., 2009) | Breast cancer | 19.1% of 1,984 patients  (denominator-received first opinion from a relevant specialist) | - Higher education level - Advised to undergo mastectomy (relative to those advised to have breast conserving surgery, or those who did not receive an advice for one procedure over another) | US |
| (Tattersall et al., 2009) | Cancer | 6.5% of 1,892 patients (denominator-received first opinion from a relevant specialist) | - Female - Younger - More educated | Australia |

**Note**: Second-opinion utilization was measured in all studies by self-reporting.
