# Supplementary material for: Seeking a second medical opinion: composition, reasons and perceived outcomes in Israel
Source: Isr J Health Policy Res. 2017 Dec 8;6:67. doi: 10.1186/s13584-017-0191-y (PMC5721599; doi:10.1186/s13584-017-0191-y)
Supplement: Supplementary file 2 — A second medical opinion survey. (DOC 54 kb) [file 13584_2017_191_MOESM2_ESM.doc]

**Appendix 2: A second medical opinion survey**

Questions to the interviewee are marked in **bold**. Instructions to the interviewer are marked in *italics*.

“Hello, my name is _________ and I am an interviewer from the Research Institute of Tel - Aviv University. I am contacting you today as part of a study on access to medical care. You were randomly selected to participate in this study. It is highly important that everyone who was selected to participate in the study would actually participate in it in order to ensure the quality of the study. Your answers will be kept confidential and will serve for the purpose of this study only. Thank you for your cooperation.”

**Section 1: Demographic details**

**How old are you______________?** *(Make sure that the interviewee is 18 years old or above)*

*For those who didn't answer the previous question:*

| **Please specify your age group?**  1 18-22  2 23-29  3 30-34  4 35-39  5 40-44  6 45-49  7 50-54  8 55-59  9 60-64  10 65-69  11 70+ |
| --- |

*(Make sure that the interviewee is 18 years old or above)*

*Interviewee’s gender?*

| 1 | Male |
| --- | --- |
| 2 | Female |

**What is your education level?**

1. Elementary school or less
2. Partial high school
3. Completed high school - without a high school diploma
4. Completed high school - with a high school diploma
5. Above high school (seminar for teachers, nursing school, technical engineering, ‘yeshiva’ (religious studies))
6. Partial academic degree – BA
7. Full academic degree - BA
8. Full academic degree - a master's degree or higher
9. *Do not read: refused to answer*

**Section 2: Second opinion utilization**

**A second medical opinion is consultation with another specialist, from the same specialty, in order to get a second opinion on the same medical concern (excluding a family physician). For example, a second opinion from an ophthalmologist after a first opinion from an ophthalmologist.**

1. **Are you aware of your right to seek a second medical opinion on a medical concern?**
2. Yes
3. No
4. Do not know
5. **Did you seek another medical opinion from another specialist, from the same specialty, in the past 12 months (excluding a family physician)?**
6. Yes
7. No → *Go to question 16*
8. *Do not read: refused to answer* → *Go to question 16*
9. **What was the specialty of the second specialist?**
10. Gynecologist
11. Orthopedist
12. Ophthalmologist
13. Dermatologist
14. Ear, Nose and Throat specialist
15. Psychiatrist
16. Cardiologist
17. General Surgeon
18. Other __________________
19. **Where did the consultation take place?**
20. Health fund clinic
21. Health fund physician in a private clinic
22. Private doctor
23. *Do not read: refuse*
24. **Did you pay for the second opinion?**
25. No
26. Yes, a fee to the specialist
27. Yes, a payment to a private doctor in an arrangement with the health fund
28. Yes, a payment to a private doctor not in any arrangement with the Health fund

*To those who visited a private physician:*

1. **What was your the main reason for visiting a private specialist and not a health fund specialist?**
   1. Waiting time at the health fund
   2. A private doctor is better/more professional than a health fund doctor
   3. Flexible hours
   4. Distance from home
   5. Confidentiality / privacy
   6. Due to the health fund restrictions to reimburse the cost of a second opinion
   7. Since a private doctor has a better attitude
   8. *Do not read: Other _____________________*
   9. *Do not read: no answer, refused*

*To those visited a private physician:*

1. **Did you claim the cost of the private second opinion from your health fund?**
   1. I claimed and did not receive a reimbursement
   2. I claimed and received a partial / full reimbursement
   3. I didn't claim

8. *Do not read: The visit was within a Consultants Network*

9. *Do not read: no answer, refused*

1. **How many specialists from the same specialty did you consult about the same medical problem (excluding your family doctor)?**
2. **What were your reasons for seeking a second medical opinion?**

*Interviewer: You can select more than one answer*

- 1. You wanted to verify a diagnosis that was given by another doctor
  2. Doubts about the recommended treatment
  3. The previous treatment was ineffective
  4. You felt that the first doctor did give you enough information about the problem and you wanted a more detailed explanation
  5. You were not satisfied with the first doctor; there was no "chemistry"
  6. You was looking for an expert in the specific domain (sub-specialty)
  7. Other reasons ________________________
  8. *Do not read: no answer, refused*

1. **What made ​​you choose the specialist from which you obtained the second opinion?**

*One answer only*

- 1. A recommendation from a friend or relative
  2. A recommendation from the health fund
  3. Information on the Internet
  4. A recommendation from the family doctor
  5. A recommendation of another consultant
  6. Previous acquaintance with the doctor within the public service
  7. Previous acquaintance with the doctor within the private service
  8. Other ___________________________
  9. *Do not read: no answer, refused*

1. **Was there a difference in diagnosis or treatment between the two specialists?**
2. Yes
3. No
4. There was a partial agreement between the diagnosis or treatment
5. *Do not read: no answer, refused*

*If yes (answer 1 or 3 question 11):*

1. **Which opinion did you choose?**
   1. First
   2. Second
   3. *Do not read: any of them*
   4. *Do not read: no answer, refused*
2. **To what extent were you satisfied with the second opinion consultation? Please rate your answer on a scale of 1 to 10 where 1 means ‘not satisfied at all’ and 10 means ‘absolutely satisfied’.**

1. **To what extent did you feel an improvement in your health condition following the second opinion consultation?**
2. Very high extent
3. High extent
4. Medium extent
5. Low extent
6. Very low extent
7. *Do not read: no answer, refused*
8. **Did you consult one of the following entities in parallel to seeking a second medical opinion, regarding the same problem?**

*Interviewer: You can select more than one answer*

- 1. Alternative Medicine
  2. Rabbi
  3. Mystical advice
  4. Psychological counseling
  5. Searched for information on the Internet
  6. *Do not read: no answer, refused*

1. **Are you a member of one of the following health funds?**
   1. Clalit
   2. Macabi
   3. Leumit
   4. Meuhedet
   5. Soldier

9. *Do not read: no answer, refused*

**Section 3: Demographic details**

**29. How would you estimate your health condition in general?**

1. Excellent
2. Very good
3. Good
4. Not so good
5. Bad

*9. Do not read: no answer, refused*

**30. Marital Status**

1. Living with a partner
2. Not Living with a partner

*9. Do not read: no answer, refused*

**31. Place of birth**

1. Born in Asia / Africa
2. Born in Europe / America
3. Born in the former Soviet Union
4. Born in Israel

*9. Do not read: no answer, refused*

**32. For those who were not born in Israel: year of immigration to Israel.**

*Please record in four digits*

**33***. (Jewish sector)* **Do you identify yourself as?**

1. Religious orthodox
2. Religious
3. Traditional – religious
4. Partially religious
5. Secular
6. Non-Jewish
7. *Do not read: between secular religious / secular believe*
8. *Do not read: Do not know / refuse to answer*

**34.** *Arab sector:* **Are you?**

1. Moslem
2. Christian
3. Druze

*9. Do not read: no answer, refused*

**35.** *Arab sector****:* Do you perceive yourself as?**

1. Very religious
2. Religious
3. Traditionally, not very religious
4. Not religious, secular
5. *Do not read: no answer, refused*

**36. The net average household income in Israel is currently about 11,000 NIS. Is your overall family income (both spouses):**

1. Well below the average
2. Slightly below average
3. As average
4. Slightly above average
5. Well above average
6. *Do not read: Not applicable - a member of Kibbutz (communal settlement)*
7. *Do not read: no answer, refused*

**37. What is the name of the city/town you live in**?

*38. Number of Statistical Area from the list?*

**Thank you for your cooperation.**
